# Neurological Outcome Following Newborn Encephalopathy With and Without Perinatal Infection: A Systematic Review

**DOI:** 10.3389/fped.2021.787804

**Published:** 2021-12-20

**Authors:** Mads Andersen, Mette Vestergård Pedersen, Ted Carl Kejlberg Andelius, Kasper Jacobsen Kyng, Tine Brink Henriksen

**Affiliations:** ^1^Department of Paediatrics and Adolescent Medicine, Aarhus University Hospital, Aarhus, Denmark; ^2^Department of Clinical Medicine, Aarhus University, Aarhus, Denmark

**Keywords:** neonatal encephalopathy, hypoxic-ischemic encephalopathy, infection, therapeutic hypothermia, neurodevelopment

## Abstract

**Background:** Studies have suggested that neurological outcome may differ in newborns with encephalopathy with and without perinatal infection. We aimed to systematically review this association.

**Methods:** We conducted this systematic review according to the Preferred Reporting Items for Systematic Reviews and Meta-Analyses (PRISMA). Studies were obtained from four databases including Pubmed, Embase, Web of Science, and The Cochrane Database. Newborns with encephalopathy with and without markers of perinatal infection were compared with regard to neurodevelopmental assessments, neurological disorders, and early biomarkers of brain damage. Risk of bias and quality of evidence were assessed by the Newcastle-Ottawa scale and Grading of Recommendations Assessment, Development and Evaluation (GRADE).

**Results:** We screened 4,284 studies of which eight cohort studies and one case-control study met inclusion criteria. A narrative synthesis was composed due to heterogeneity between studies. Six studies were classified as having low risk of bias, while three studies were classified as having high risk of bias. Across all outcomes, the quality of evidence was very low. The neurological outcome was similar in newborns with encephalopathy with and without markers of perinatal infection.

**Conclusions:** Further studies of higher quality are needed to clarify whether perinatal infection may affect neurological outcome following newborn encephalopathy.

**Systematic Review Registration:** https://www.crd.york.ac.uk/prospero/, identifier CRD42020185717.

## Introduction

Neonatal encephalopathy due to intrapartum-related events–newborn encephalopathy–is a major contributor to infant mortality and neurodevelopmental morbidity (1). When hypoxia-ischemia is the suspected cause of newborn encephalopathy, therapeutic hypothermia may be applied as neuroprotective treatment (2). However, some 50% of all newborns treated with hypothermia still develop unfavorable neurological outcomes (2, 3).

While hypoxia-ischemia may be commonly involved in newborn encephalopathy, the etiology remains multifactorial (4, 5). Perinatal infection is also associated with newborn encephalopathy (6–14). Ascending bacteria may spread to the fetus or result in fetal inflammatory response syndrome, characterized by systemic and local inflammation in the fetal organs including the brain (15, 16). Bacteria from the birth canal may also be transmitted during delivery leading to early-onset infection with similar activation of the newborn inflammatory system (17). Infection and inflammation in the central nervous system may cause encephalopathy and brain damage *per se* or act in combination with other insults such as hypoxia-ischemia (18–20). This *multiple-hit hypothesis* states that one insult could sensitize the fetal or newborn brain in order for secondary insults to have larger clinical impact (20–23). Several animal studies have found that endotoxin-induced inflammation prior to hypoxia-ischemia severely exacerbates brain injury in newborns (24–31). In addition to the possible aggravation of brain damage, both animal (32–37) and clinical studies (38–41) have suggested that infections may affect both the efficacy and safety of therapeutic hypothermia.

Accordingly, perinatal infection may interfere with the prognosis in newborns with encephalopathy. A systematic review of this association in humans may qualify the need for differential treatment in newborns with encephalopathy born by mothers with infection or with infection themselves. Therefore, the aim of this systematic review was to compare newborns with encephalopathy with and without markers of perinatal infection with regard to neurological outcome including neurodevelopmental assessments, neurological disorders, and early biomarkers of brain damage.

## Methods

This systematic review was performed in accordance with the Preferred Reporting Items for Systematic Reviews and Meta-analyses (PRISMA) (Supplementary A) (42). The protocol was registered 12^th^ of May 2020 at the International Prospective Register of Systematic Reviews (PROSPERO) (CRD42020185717) (43). Two authors (MA and MVP) independently screened articles, assessed eligibility, extracted data, and analyzed risk of bias and quality of evidence. Any disagreements were resolved by discussion or by a third reviewer (TBH).

### Terminology

Neonatal encephalopathy is a broad term including different etiologies, while hypoxic-ischemic encephalopathy may be used when hypoxia-ischemia is the most likely cause of the encephalopathy (44, 45). In this systematic review, we used the term “newborn encephalopathy” to emphasize our interest in insults surrounding the birth and the uncertainty with underlying causes. However, when discussing the individual studies, we continued the use of their specific terminology. In addition, no consensus definition of neonatal sepsis currently exists (46). Positive blood culture remains the golden standard of diagnosis. However, several neonates are believed to have sepsis without ever having isolated the specific pathogen (47, 48). Again, we opted to continue the terminology and definition of sepsis used in each individual study.

### Eligibility Criteria

#### Studies

We only included peer-reviewed studies. Both observational studies and randomized controlled trials were eligible for inclusion, while case reports and case series were excluded. No restrictions by language or publication dates were applied.

#### Population

Newborns with a gestational age of ≥36 completed weeks or with a birth weight of ≥2500 g.

#### Exposure

Newborns with a diagnosis of neonatal encephalopathy or hypoxic-ischemic encephalopathy in combination with markers of bacterial infection in the mother or child during the perinatal period. Markers of maternal infection included clinical or histological chorioamnionitis, funisitis, and/or chorionic vasculitis. Markers of early-onset infection included infection proven by culture or molecular testing or suspected infection as assessed by the clinician based on clinical features, decision to initiate and continue antimicrobial treatment, and biomarkers including either white blood-cell counts, neutrophil counts, ratio of immature to total neutrophils, C-reactive protein, procalcitonin, or interleukins (IL-6, IL-8, or IL-10).

#### Comparators

Newborns with a diagnosis of neonatal encephalopathy or hypoxic-ischemic encephalopathy without the markers of bacterial infection in the mother or child during the perinatal period.

#### Outcome

The primary outcome was the composite outcome of (1) scales and tools for assessing neurodevelopmental function, (2) neurological disorders including seizure-, motor-, cognitive-, mental-, and behavioral disorders, and (3) mortality. The secondary outcomes were defined as the above outcomes assessed separately except for mortality. Furthermore, we included biomarkers of early brain damage such as magnetic resonance imaging (MRI) measures and MR spectroscopy, as well as conventional and amplitude-integrated electroencephalography (49, 50).

### Information Sources and Search Strategy

The search strategy was developed by the authors and tested before use. The primary search was conducted 12 April 2021. Pubmed, Embase, Web of Science, and The Cochrane Database were searched by use of subject headings and free texts related to newborns, encephalopathy, and infection (Supplementary B). Our search was limited to human studies. References and citations from each included study were manually scrutinized for additional relevant studies.

### Study Selection

The search results from each database were pooled using Endnote X9® and duplicates were removed. Rayyan QCRI was used in the screening process (51). Studies found by search were screened by title and abstract, while references and citations from included studies were screened by title only. All studies that seemingly met the eligibility criteria or provided insufficient information were extracted for full-text analysis. If any information regarding the eligibility criteria was missing, the study was excluded (Supplementary C).

### Data Collection and Data Items

Data were extracted by a predefined data-collection form. To reduce errors and missing data plots, the data form was piloted prior to data extraction. Following items were extracted from each study: (1) title, authors, country, journal, year of publication, references, citations, funding sources, and conflicts of interest; (2) aim of the study, design, setting, and time-period; (3) number and characteristics of the newborns; (4) assessments of encephalopathy and whether the newborns received therapeutic hypothermia; (5) assessments of maternal and newborn infections; (6) assessments of neurological outcome; and (7) statistical methods and results (Supplementary D).

### Risk of Bias in Individual Studies

The Newcastle-Ottawa Scale for cohort studies was used to evaluate the risk of bias. The studies were evaluated on the risk of bias in the domains: selection process, comparability between groups, and assessment of outcome. A total of four, two, and three points could be awarded in each domain, respectively. The risk of bias in the studies was rated as “low” when awarded 3–4 points in selection, 1–2 points in comparability, and 2–3 points in outcome; “fair” when awarded 2 points in selection, 1-2 points in comparability, and 2–3 points in outcome; and “high” when awarded 0–1 point in selection or 0 point in comparability or 0–1 point in outcome. When assessing comparability, one point was awarded if the study controlled for malformations. An additional point was awarded if the study controlled for gestational age, birth weight, gender, or metabolic diseases.

### Synthesis of Results

No meta-analysis was performed due to the heterogeneity between the studies with regard to the assessment of encephalopathy, infection, and neurological outcome. A narrative synthesis of the results was made in accordance with Popay et al. (52). In studies with inadequate summary measures, a Chi-square test or Fischer's exact test was performed to assess odds ratio with 95% confidence interval. Analyses were performed by GraphPad Prism version 8.0.0 for Mac (GraphPad Software, San Diego, California USA, www.graphpad.com).

### Quality of Evidence

The quality of evidence was assessed in accordance with GRADE (53, 54). A total of five domains were evaluated including the risk of bias, directness of evidence, heterogeneity, precision of effect estimates, and risk of publication bias. Due to their observational design, the studies started with an initial rating of low quality. The studies were then downgraded for serious limitations in any of the five domains and would only be upgraded if no downgrading had occurred. Accordingly, the quality of evidence could be rated as high, moderate, low, or very low.

### Risk of Bias Across Studies

Publication bias was assessed qualitatively based on the characteristics of the included studies as an insufficient number of studies was included for formal test of asymmetry. Selective reporting bias was assessed by comparing the outcomes reported in the method section and the result section of the included studies.

## Results

### Study Selection

**Figure 1:**
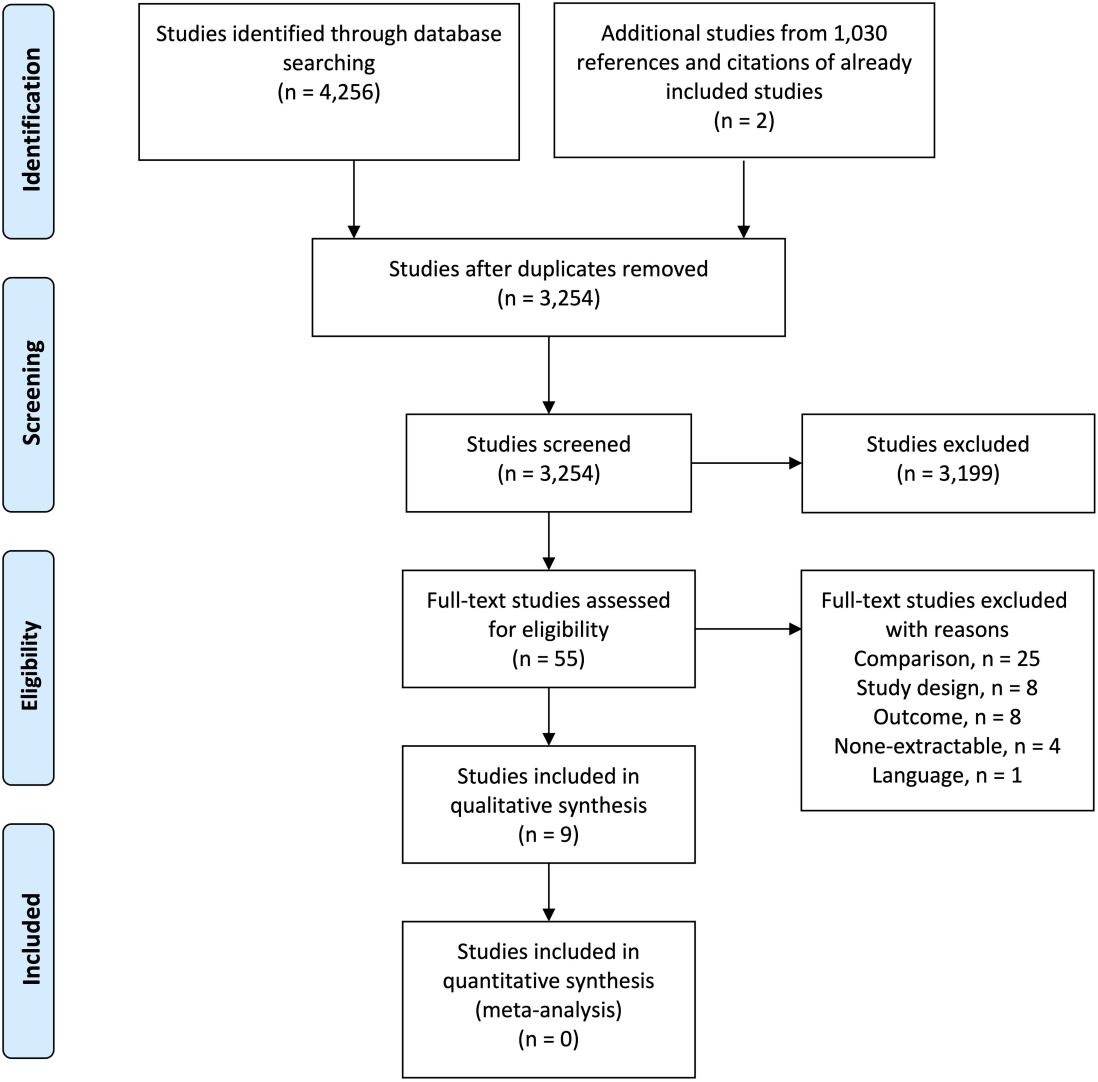
PRISMA flow diagram of the study selection process.

A total of 4,256 studies were identified across all databases. After removal of duplicates, 3,254 studies were screened for inclusion. Of these, 55 studies were evaluated by full-text and seven studies were eligible for inclusion. Two additional studies were included following screening of 1,030 references and citations from already included studies. This led to inclusion of nine studies for this systematic review (55–63). An overview of the selection process is illustrated in the PRISMA flow diagram (Figure 1).

### Study Characteristics

#### Designs and Populations

Eight cohort studies (55–61, 63) and one case-control study (62) were included. Table 1 provides number, gestational age, birth weight, and gender of the newborns, separated by infection status when possible. Three studies from the Netherlands have overlapping populations (56, 57, 61). Hakobyan et al. (56) only included historical controls (64). Newborns with encephalopathy fulfilled criteria for therapeutic hypothermia in seven studies (55–60, 63), while hypothermia was introduced midtrial in a single study (61). One study was conducted before the implementation of hypothermia (62).

**Table 1:** Number, gestational age, birth weight, and male to female (M/F) ratio of the newborns in the studies investigating the association between newborn encephalopathy, perinatal infection, and neurological outcome.

**Study and country**	**Groups**	**Number**	**Gestational age (weeks)**	**Birth weight (g)**	**M/F-ratio**
Rao (55)^a^ USA	Proven infection Probable infection No infection	36 255 1,243	40 (39–40) 39 (38–40) 39 (38–40)	3,471 (3,075–3,835) 3,370 (2,990–3,771) 3,260 (2,911–3,670)	22/14 145/110 693/550
Hakobyan (56)^b^ Netherlands	Proven sepsis Probable sepsis No infection	14 28 308	40 (1.7) 40 (1.5) 40 (1.6)	3,658 (534) 3,660 (726) 3,401 (612)	7/7 14/14 164/144
Frank (57)^a^ Netherlands	All neonates	76	40 (36–42)	3,455 (2,100–5,700)	41/35
Orrock (58)^d^ USA	All neonates	28	>36	>1,800	NDA
Mir (59)^b^ USA	All neonates	73	39 (2)	3,384 (607)	NDA
Lachapelle (60)^b^ Canada	All neonates	103	39 (1.5)	3,411 (662)	58/45
Harteman (61)^c^ Netherlands	All neonates	95	40 (36–42)	3,290 (2,030–5,500)	54/41
Hayes (62)^d^ Ireland	All neonates	56	>36	NDA	NDA
Wintermark (63)^b^ Canada	All neonates	23	39 (1.2)	3,385 (408)	13/10

*NDA, no data available*.

*Gestational ages and birth weights presented as*

a *median (lower to upper quartile)*,

b *mean (standard deviation)*,

c *median (range), and*

d *based on inclusion criteria*.

#### Markers of Hypoxia-Ischemia

Table 2 provides the potential markers of hypoxia-ischemia, which was part of the inclusion criteria in each study. Six studies stated that they included newborns with encephalopathy and biochemical criteria suggestive of hypoxia-ischemia including low umbilical arterial pH and high base deficit (55, 58–61, 63). The occurrence of perinatal sentinel events as potential causes of hypoxia-ischemia was part of the criteria in five studies; however, the occurrence was not fully necessary for inclusion (55, 58–60, 63). Three studies solely stated that they included newborns with encephalopathy with signs of asphyxia or due to presumed hypoxia-ischemia (56, 57, 62). However, biochemical markers and other signs of fetal and neonatal distress were presented in the descriptive data of the studies.

**Table 2:** Possible markers of hypoxia-ischemia as part of inclusion criteria in the studies investigating the association between newborn encephalopathy, perinatal infection, and neurological outcome.

**Study**	**Used terminology**	**Therapeutic hypothermia**	**Biochemical markers**	**Other evidence of neonatal distress^**a**^**	**Other evidence of fetal distress^**a**^**	**Multiorgan failure^**a**^**
Rao (55)	Hypoxic-ischemic encephalopathy	Yes	Arterial cord pH ≤7 or BD ≥16 If unavailable or pH was between 7.01 and 7.15 or BD between 10 and 16, additional criteria were needed	Clinical encephalopathy Seizures Apgar score ≤5 Continuous need for ventilation Abnormal aEEG Neuroimaging of ischemia within 7 days of life	History of acute perinatal event Evidence of fetal distress (heart rate monitoring, umbilical cord Doppler, or biophysical profile)	Yes
Hakobyan (56)^b^	Neonatal encephalopathy with signs of asphyxia	Yes				
Frank (57)^b^	Neonatal encephalopathy with signs of asphyxia	Yes		Clinical encephalopathy Abnormal aEEG		
Orrock (58)	Hypoxic-ischemic encephalopathy	Yes	Arterial cord pH ≤7 or BD ≥16 If unavailable or pH was between 7.01 and 7.15 or BD between 10 and 15.9, additional criteria were needed	Clinical encephalopathy Seizures Apgar score ≤5 Continuous need for ventilation	History of acute perinatal event	
Mir (59)	Neonatal encephalopathy	Yes	Arterial cord pH ≤7 or BD ≥16 If unavailable or pH was between 7.01 and 7.15 or BD between 10 and 15.9, additional criteria were needed	Clinical encephalopathy Apgar score ≤5 Continuous need for ventilation	History of acute perinatal event	
Lachapelle (60)	Neonatal encephalopathy with signs of asphyxia	Yes	Arterial cord pH ≤7 or BD ≥16 Possibly blood gas <1 h of life with pH ≤7 or BD ≥16	Clinical encephalopathy Apgar score ≤5 Continuous need for ventilation Abnormal aEEG	History of acute perinatal event	
Harteman (61)	Neonatal encephalopathy after presumed hypoxia-ischemia	Midtrial	Arterial cord pH <7.1	Clinical encephalopathy Seizures Delayed onset of respiration Apgar score ≤7 Thompson score >7	Late decelerations on fetal monitoring	Yes
Hayes (62)^b^	Neonatal encephalopathy after presumed hypoxia-ischemia	No				
Wintermark (63)	Hypoxic-ischemic encephalopathy	Yes	Possibly arterial cord pH ≤7 or BD ≥16 Possibly blood gas <1 h of life with pH ≤7 or BD ≥16	Clinical encephalopathy Apgar score ≤5 Continuous need for ventilation Abnormal aEEG	History of acute perinatal event Biophysical profile	Yes

*BD, base deficit; aEEG, amplitude-integrated electroencephalogram*.

a *Not all criteria fully necessary for inclusion*,

b *Presence of biochemical markers and evidence of fetal and neonatal distress was presented in the descriptive data of the studies*.

#### Markers of Perinatal Infection

Seven studies investigated newborns born by mothers with markers of infection (57–63). Six studies stated that histological chorioamnionitis, funisitis, or chorionic vasculitis were assessed by a blinded pathologist (57, 59–63), while one study stated that the diagnoses were obtained from placental pathology reports (58). Two studies investigated newborns with markers of infection (55, 56). Rao et al. (55) identified infections within seven days of life. Proven infection was defined by a positive culture (blood, urine, cerebrospinal fluid, airways), while suspected newborns had negative cultures but still received antimicrobial treatment for 7 to 10 days. Hakobyan et al. (56) included newborns who all showed clinical signs of sepsis within 48 h. In addition, the newborns had positive blood cultures, positive surface cultures, or elevated C-reactive protein (≥50 mg/L). All newborns received antimicrobial treatment for at least 7 days.

#### Neurological Outcomes

Tables 3, 4 provide timing of neurological assessment in each study, which spanned from soon after birth to around 24 months of life. Neurodevelopmental outcomes were investigated in three studies (56, 57, 59). Hakobyan et al. and Frank et al. (56, 57) defined adverse neurological outcome as death, neurological disability (e.g., cerebral palsy), or abnormal Griffiths' neurodevelopmental quotient (<88 or 85) or Bayley Scales of Infant and Toddler Development-III (Bayley-III) score (<85). Mir et al. (59) also investigated a composite outcome consisting of death or low Bayley-III score (<85). MRI findings were investigated in seven studies (55, 57, 58, 60–63). Rao et al. (55) obtained MRI findings classified as either normal, abnormal, cortical injury, deep gray matter injury, or white matter injury. Frank et al. and Harteman et al. (57, 61) classified patterns of brain injury by MRI as normal, white matter/watershed injury, basal-ganglia-thalamus injury, or injury in both. Classification in similar manner based on Barkovich et al. (65) was used in three studies (58, 60, 62). Wintermark et al. (63) identified MRI findings showing evidence of hypoxic-ischemic brain injury. Two studies used postmortem brain examinations or cerebral ultrasound when MRI was unavailable (57, 60).

**Table 3:** Odds ratios (OR) with 95% confidence intervals (CI) of unfavorable neurological outcome between newborns with encephalopathy with and without maternal infection.

**Study**	**Exposure**	**Adverse outcome**	**Follow-up**	**Exposed**	**Comparators**	**OR (95% CI)**
Frank (57)	Chorioamnionitis Funisitis	**Death, cerebral palsy, or Griffiths' neurodevelopment quotient <85 or Bayley-III <85**	18–24 months of age	11/29 (38 %)	24/47 (51 %)	0.6 (0.2–1.4)
				4/12 (33 %)	31/64 (48 %)	0.5 (0.2–1.8)
Mir (59)	Chorioamnionitis Chorioamnionitis with fetal response	**Death or Bayley-III score <85**	18–24 months of age	26/47 (55 %)	14/26 (54 %)	1.1 (0.7–2.8)
				14/32 (44 %)	26/41 (63 %)	0.4 (0.2–1.1)
Frank (57)	Chorioamnionitis Funisitis	MRI evidence of WM/WS, BGT, or near total injury	NDA	15/29 (52 %)	33/46 (72 %)	0.4 (0.2–1.1)
				5/12 (42 %)	43/63 (68 %)	0.3 (0.1–1.2)
Orrock (58)	Chorioamnionitis	Died or MRI evidence of WM/WS or BGT damage	10–12 days of age	3/9 (33 %)	9/19 (47 %)	0.6 (0.1–2.9)
Lachapelle (60)	Chorioamnionitis with or without vasculitis	MRI evidence of WM/WS or BGT damage		12/30 (40 %)	41/73 (56 %)	0.5 (0.2–1.2)
Harteman (61)	Chorioamnionitis Funisitis	MRI evidence of injury in WM/WS, BGT, or WM/WS with BGT involvement	2–15 days of age	24/44 (55 %)	37/51 (73 %)	0.5 (0.2–1.0)
				13/23 (56 %)	48/72 (67 %)	0.7 (0.2–1.6)
Hayes (62)	Funisitis Vasculitis	MRI evidence of injury in WS, BG, both, or other brain injuries	Before 1 month of age	1/3 (33 %)	34/59 (58 %)	0.4 (0.1–3.3)
				12/19 (63 %)	22/37 (59 %)	1.2 (0.4–3.5)
Wintermark (63)	Chorioamnionitis	MRI evidence of hypoxic-ischemic brain injury	NDA	4/8 (50 %)	3/15 (20 %)	4.0 (0.7–20.4)
	Chorioamnionitis with vasculitis			3/5 (60 %)	4/18 (22 %)	6.0 (0.8–41.4)

*NDA, no data available; MRI, magnetic resonance imaging; WM/WS, white matter/watershed; BGT, basal-ganglia-thalamus; Bayley-III, Bayley Scales of Infant and Toddler Development-III*.

*Odds ratios with 95% confidence intervals were calculated by Fisher's exact test. Adverse outcomes in bold indicate our primary outcomes*.

**Table 4:** Odds ratios (OR) with 95% confidence intervals (CI) of unfavorable neurological outcome in newborns with encephalopathy with and without early-onset infection.

**Study**	**Exposure**	**Adverse outcome**	**Follow-up**	**Exposed**	**Comparators**	**OR (95% CI)**
Hakobyan (56)	Proven or probable sepsis	**Death, cerebral palsy, neurodevelopmental impairment of** **>3 months, Griffith's neurodevelopment quotient <88, or Bayley-III <85**	At least 18 months	14/42 (33 %)	140/308 (45 %)	0.6 (0.3–1.2)
Rao (55)	Suspected infection	Abnormal MRI findings Cortical injury on MRI	NDA	31/255 (12 %)	155/1,243 (12 %)	1.0 (0.6–1.4)
				29/255 (11 %)	140/1,243 (11 %)	1.0 (0.7–1.5)
		Deep gray matter injury on MRI		49/255 (19 %)	197/1,243 (16 %)	1.3 (0.9–1.8)
		White matter injury on MRI		46/255 (18 %)	166/1,243 (13 %)	1.4 (1.0–2.0)
		Normal MRI findings		78/255 (30 %)	404/1,243 (33 %)	0.9 (0.7–1.2)
	Confirmed infection	Abnormal MRI findings Cortical injury on MRI		8/36 (22 %)	155/1,243 (12 %)	2.0 (0.9–4.4)
				4/36 (11 %)	140/1,243 (11 %)	1.1 (0.4–3.0)
		Deep gray matter injury on MRI		8/36 (22 %)	197/1,243 (16 %)	1.5 (0.7–3.2)
		White matter injury on MRI		5/36 (14 %)	166/1,243 (13 %)	1.0 (0.4–2.6)
		Normal MRI findings		10/36 (28 %)	404/1,243 (33 %)	0.8 (0.4–1.7)

*NDA, no data available; MRI, magnetic resonance imaging; Bayley III, Bayley Scales of Infant and Toddler Development-III*.

*Odds ratios with 95% confidence intervals were calculated by Chi-square test or Fisher's exact test. Adverse outcomes in bold indicate our primary outcomes*.

### Risk of Bias Within Studies

Table 5 provides an overview of the points awarded by the Newcastle-Ottawa Scale. The studies failed to report on several of the selected risk factors for quality assessment and three studies failed to report on any of these (59, 60, 63). Rao et al. (55) excluded newborns with major congenital anomalies and found no difference between newborns with and without early-onset infection with regard to gestational age and gender, but observed that newborns with infections had higher birth weights. Hakobyan et al. (56) found no difference between newborns with and without early-onset sepsis with regard to gestational age, birth weight, and gender. Orrock et al. (58) excluded newborns with major congenital abnormalities. Three studies excluded newborns with either chromosomal or metabolic disorders (57, 61, 62). In total, six studies were classified as having low risk of bias (55–58, 61, 62), while three studies were classified as having high risk of bias (59, 60, 63).

**Table 5:** Points awarded by the Newcastle-Ottawa Scale to the included studies investigating the association between newborn encephalopathy, perinatal infection, and neurological outcome.

**Study**	**Representativeness of exposed cohort^**a**^**	**Selection of non-exposed^**b**^**	**Ascertainment of exposure^**c**^**	**Presences of outcome of interest^**d**^**	**Comparability^**e**^**	**Assessment of outcome^**f**^**	**Enough follow-up^**g**^**	**Adequacy of follow up^**h**^**	**Risk of bias**
Rao (55)	A ( 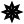 )	A ( 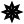 )	A ( 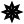 )	B ( 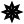 )	A, B ( 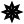 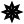 )	B ( 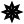 )	A ( 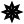 )	D	Low
Hakobyan (56)	A ( 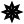 )	B	A ( 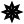 )	A ( 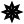 )	B ( 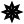 )	B ( 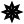 )	A ( 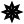 )	B ( 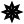 )	Low
Frank (57)	B ( 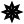 )	A ( 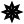 )	A ( 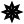 )	A ( 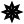 )	A, B ( 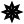 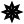 )	B/D ( 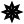 )^i^	A ( 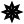 )	A ( 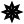 )	Low
Orrock (58)	C	A ( 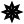 )	A ( 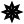 )	A ( 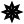 )	A ( 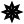 )	A ( 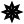 )	A ( 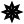 )	A ( 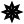 )	Low
Mir (59)	A ( 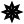 )	A ( 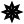 )	A ( 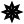 )	A ( 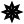 )		A ( 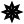 )	A ( 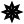 )	A ( 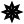 )	High
Lachapelle (60)	B ( 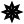 )	A ( 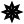 )	A ( 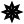 )	A ( 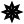 )		A ( 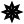 )	A ( 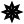 )	A ( 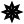 )	High
Harteman (61)	B ( 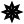 )	A ( 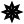 )	A ( 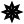 )	A ( 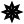 )	A, B ( 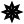 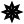 )	D	A ( 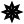 )	A ( 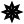 )	Low
Hayes (62)	B ( 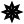 )	A ( 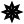 )	A ( 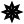 )	A ( 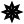 )	A, B ( 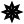 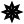 )	A ( 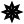 )	A ( 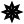 )	C	Low
Wintermark (63)	A ( 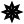 )	A ( 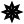 )	A ( 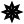 )	A ( 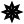 )		B ( 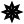 )	A ( 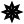 )	A ( 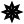 )	High

Studies were rated as “low risk” when given 3–4 points (*) in selection and 1–2 points (*) in comparability and 2–3 points (*) in outcome; “fair risk” when given 2 points (*) in selection and 1–2 points (*) in comparability and 2–3 points (*) in outcome; and “high risk” when given 0–1 point (*) in selection or 0 point (*) in comparability or 0–1 point (*) in outcome.

a *Representativeness of the exposed cohort, a) truly representative, b) somewhat representative, c) selected group, and d) no description*;

b *Selection of the non-exposed cohort, a) drawn from the same community, b) drawn from a different source, and c) no description*;

c *Ascertainment of exposure, a) secure record, b) structured interview, c) written self-report, and d) no description*;

d *Demonstration that outcome of interest was not present at start of study, a) yes and b) no*;

e *Comparability of cohorts or cases and controls, a) study controls for malformation and b) study controls for either metabolic diseases, gestational age, birth weight, or gender*;

f *Assessment of outcome, a) independent blind assessment, b) record linkage, c) self-report, and d) no description*;

g *Follow-up long enough for outcomes to occur, a) yes and b) no*;

h *Adequacy of follow up of cohorts, a) complete follow up, b) subjects lost to follow up unlikely to introduce bias, >5% follow up or description provided of those lost, c) follow up rate <5% and no description of those lost, and d) no statement*;

i *B for assessment of long-term neurodevelopmental outcome and D for assessment of magnetic resonance imaging*.

### Results of Individual Studies

#### Maternal Infection

A summary of the results is presented in Table 3. The studies found no statistically significant difference in neurological outcome between newborns with encephalopathy born by mothers with and without markers of infection (57–63). Frank et al. and Mir et al. (57, 59) found tendencies toward more favorable neurodevelopment in newborns born by mothers with chorioamnionitis or funisitis. With regard to neuroimaging, most studies also reported tendencies toward more favorable MRI findings in newborns with maternal infections (57, 58, 60–62).

#### Early-Onset Infection

A summary of the results is presented in Table 4. Hakobyan et al. (56) found no difference in death or unfavorable neurodevelopment between newborns with neonatal encephalopathy with and without markers of early-onset sepsis. Rao et al. (55) similarly found no difference in MRI findings between newborns with hypoxic-ischemic encephalopathy with and without proven or suspected early-onset infections.

#### Therapeutic Hypothermia

The studies found no statistically significant difference between hypothermia-treated newborns with encephalopathy with and without markers of perinatal infection (55–60, 63).

### Quality of Evidence

We found that the quality of evidence across all outcomes was very low according to the GRADE assessment. This was mainly due to a concern related to risk of bias within the studies and the small number of included newborns. Furthermore, early MRI findings as proxy measures for neurodevelopment subjected the studies to downgrading for indirectness (53, 54).

### Risk of Bias Across Studies

Selective reporting bias was not identified. Several of the studies reported no difference in neurological outcome between newborns with and without markers of perinatal infections. Furthermore, the association between perinatal infections and neurological outcome was not the primary aim in several studies (55, 57–63). Therefore, publication bias seems improbable.

## Discussion

### Summary of Evidence

Based on the included studies, the presence of perinatal infection does not seem to impact neurological outcome in newborns with encephalopathy. However, the quality of evidence was very low.

Markers of maternal infection have been associated with newborn encephalopathy (6–12). However, we found no further association with worse neurological outcome. Some studies on maternal infection were excluded from this systematic review, despite having some relevance (Supplementary C). Nelson et al. (66) found that the combination of perinatal sentinel events and markers of maternal infection was associated with an increased risk of cerebral palsy compared with perinatal sentinel events alone. Jenster et al. (67) investigated newborns with clinical and biochemical evidence of hypoxia-ischemia including 5-min Apgar score ≤5 and arterial cord pH <7.1 and base deficit >10. By contrast, they found that chorioamnionitis was associated with more favorable MRI findings at around 5 days of age and more favorable neurodevelopment assessed by Bayley-II or III at around 30 months of age. Early-onset infections may be more prevalent in newborns with encephalopathy and are associated with increased mortality (13, 14). However, as with maternal infections, we found no difference in neurological outcome between newborns with encephalopathy with and without early-onset infection. Both included studies on early-onset infections had a limited number of newborns with positive cultures. Hakobyan et al. (56) analyzed the combined number of newborns with documented and suspected sepsis. Only a smaller fraction (<1/3) considered to have an infection had positive culture, making the diagnosis less well defined. Furthermore, the study only included historical controls, which further limits the quality of evidence. Again, some studies on early-onset infection with some relevance for the topic were excluded (Supplementary C). Scheidegger et al. (68) found no difference in the Sarnat Staging during the first days of life between newborns with hypoxic-ischemic encephalopathy with and without early-onset sepsis (69). However, Jenster et al. (67) found early-onset sepsis to be associated with worse neuromotor function at around 30 months of age in newborns with clinical and biochemical markers of hypoxia-ischemia. In addition, when combining newborns with encephalopathy treated with and without hypothermia from a recent randomized controlled trial in low- and middle-income countries, culture-positive early- and late-onset sepsis were associated with increased risk of death or disability at 18 months (39).

Animal studies have suggested that infectious and inflammatory exposure before hypoxia-ischemia exacerbate newborn brain injury (24–31). Several biological mechanisms have been suggested to explain these findings (70–86). However, this systematic review of human studies was not able to substantiate these findings. This may be due to more heterogeneity inherent in clinical compared to experimental studies. A previous study found perinatal sentinel events, as potential causes of hypoxia-ischemia, only to be present in some 15% of newborns with encephalopathy (8). The timing between different perinatal insults may also have influenced the neurological outcome. Animal studies have found that lipopolysaccharides from *Escherichia coli* administered 4 h, 6 h, and 72 h before hypoxia-ischemia result in more severe brain damage, while lipopolysaccharides administered 24 h before hypoxia-ischemia had neuroprotective effects (24, 25, 86, 87). Therefore, both positive and negative conditioning may occur in the fetal or newborn brain when exposed to multiple insults (88). This may explain the findings of the included study by Harteman et al. (61), who found that newborns with hypoxic-ischemic encephalopathy born by mothers with chorioamnionitis had the highest incidence of both normal and most severe MRI findings.

Several newborns with encephalopathy due to presumed hypoxia-ischemia still develop unfavorable neurological outcomes despite treatment with therapeutic hypothermia (2, 3). Several reviews have postulated that therapeutic hypothermia may be contraindicated in newborns with encephalopathy and infection (19, 20, 89, 90). This systematic review found similar neurological outcome between hypothermia-treated newborns with encephalopathy with and without markers of perinatal infection (55–60, 63). However, due to the very low quality of evidence across all outcomes, this concern may still be valid. To our knowledge, no randomized clinical trial has sufficiently investigated the neuroprotective effect of hypothermia in newborns with encephalopathy and perinatal infection, although no association between hypothermia and the risk of sepsis has been found (2). Therapeutic hypothermia has been shown to delay C-reactive protein response and to suppress white blood-cell count (91, 92). These findings may partly explain the reduced efficacy of hypothermia observed in low- and middle-income countries (where infections may be more prevalent) (38, 39), and the increased risk of mortality and prolonged shock observed in hypothermia-treated adults with meningitis and sepsis (40, 41). Furthermore, animal studies have found hypothermia to have limited neuroprotective effect following lipopolysaccharide-sensitized hypoxia-ischemia (32–37, 93). Both studies on early-onset infections included in this systematic review reported an overrepresentation of Gram-positive bacteria (55, 56). Contrarily, animal studies have found hypothermia to have some neuroprotective effect following sensitization by endotoxins deriving from Gram-positive bacteria (34, 94).

### Strengths and Limitations

We conducted this systematic review according to the PRISMA guidelines (42). We followed a preregistered protocol. We conducted a systematic search across four different databases. To minimize bias, each step was performed independently by two reviewers including screening of studies, data collection, and risk of bias and quality of evidence assessment. However, several limitations have to be considered. Newborn encephalopathy may arise from different etiologies including hypoxia-ischemia, infection and inflammation, placental pathologies, and more (5). These factors may alone or together affect the fetal or newborn brain. In the included studies, newborn encephalopathy may have occurred due to various factors and interactions. However, most studies presented criteria suggestive of both hypoxia-ischemia and perinatal infection, indicating that these factors were involved to some degree. The assessments of neurological outcomes may also be problematic. Most studies reported MRI findings, which not necessarily correlates with the neurodevelopmental outcome (95, 96). Furthermore, the longest follow-up time in the included studies was around 24 months of age. It would have been interesting to observe whether any differences in neurological outcome would develop throughout childhood and adolescence (97). Furthermore, due to inadequate reporting in several studies, we were unable to reject that the reference groups also contained mothers or newborns exposed to perinatal infections. This have likely biased the studies toward findings of no difference. At last, most included studies also contained small number of newborns and the comparability between newborns with and without perinatal infections was limited. This led to imprecision of the effect sizes and a high possibility of risk of bias. A meta-analysis could have increased the precision; however, the included studies were deemed to heterogenous.

## Conclusion

We found no difference in neurological outcome between newborns with encephalopathy with and without markers of perinatal infection. However, the current quality of evidence within this subject is very low. Therefore, further studies are needed with larger sample sizes, longer follow-up time, less risk of bias, and more detailed description of populations with reports of possible etiologies and interactions.

## Data Availability Statement

The original contributions presented in the study are included in the article/Supplementary Material, further inquiries can be directed to the corresponding author.

## Author Contributions

MA, MP, TA, KK, and TH designed the study. MA and MP undertook data collection and analysis. MA drafted the manuscript. All authors have critically reviewed the drafted manuscript and have approved the manuscript and agree to be accountable for all aspects of the work.

## Funding

This study was supported by Aarhus University (Graduate School of Health) and The Elsass Foundation (19-3-0577).

## Conflict of Interest

The authors declare that the research was conducted in the absence of any commercial or financial relationships that could be construed as a potential conflict of interest.

## Publisher's Note

All claims expressed in this article are solely those of the authors and do not necessarily represent those of their affiliated organizations, or those of the publisher, the editors and the reviewers. Any product that may be evaluated in this article, or claim that may be made by its manufacturer, is not guaranteed or endorsed by the publisher.

## References

[1] Lee AC Kozuki N Blencowe H Vos T Bahalim A Darmstadt GL . Intrapartum-related neonatal encephalopathy incidence and impairment at regional and global levels for 2010 with trends from 1990. Pediatr Res. (2013) 74 Suppl 1:50–72. 10.1038/pr.2013.206 24366463 PMC3873711

[2] Jacobs SE Berg M Hunt R Tarnow-Mordi WO Inder TE Davis PG. Cooling for newborns with hypoxic ischaemic encephalopathy. Cochrane Database Syst Rev. (2013) (1):CD003311. 10.1002/14651858.CD003311.pub3 23440789 PMC7003568

[3] Edwards AD Brocklehurst P Gunn AJ Halliday H Juszczak E Levene M . Neurological outcomes at 18 months of age after moderate hypothermia for perinatal hypoxic ischaemic encephalopathy: synthesis and meta-analysis of trial data. BMJ. (2010) 340:c363. 10.1136/bmj.c363 20144981 PMC2819259

[4] Kurinczuk JJ White-Koning M Badawi N. Epidemiology of neonatal encephalopathy and hypoxic-ischaemic encephalopathy. Early Hum Dev. (2010) 86:329–38. 10.1016/j.earlhumdev.2010.05.010 20554402

[5] Aslam S Strickland T Molloy EJ. Neonatal encephalopathy: need for recognition of multiple etiologies for optimal management. Front Pediatr. (2019) 7:142. 10.3389/fped.2019.00142 31058120 PMC6477286

[6] Badawi N Kurinczuk JJ Keogh JM Alessandri LM O'Sullivan F Burton PR . Intrapartum risk factors for newborn encephalopathy: the Western Australian case-control study. BMJ. (1998) 317:1554–8. 10.1136/bmj.317.7172.1554 9836653 PMC28733

[7] Impey L Greenwood C MacQuillan K Reynolds M Sheil O. Fever in labour and neonatal encephalopathy: a prospective cohort study. BJOG. (2001) 108:594–7. 10.1111/j.1471-0528.2001.00145.x 11426893

[8] Nelson KB Bingham P Edwards EM Horbar JD Kenny MJ Inder T . Antecedents of neonatal encephalopathy in the Vermont Oxford Network Encephalopathy Registry. Pediatrics. (2012) 130:878–86. 10.1542/peds.2012-0714 23071210 PMC4074646

[9] Tann CJ Nakakeeto M Willey BA Sewegaba M Webb EL Oke I . Perinatal risk factors for neonatal encephalopathy: an unmatched case-control study. Arch Dis Child Fetal Neonatal Ed. (2018) 103:F250–6. 10.1136/archdischild-2017-312744 28780500 PMC5916101

[10] Blume HK Li CI Loch CM Koepsell TD. Intrapartum fever and chorioamnionitis as risks for encephalopathy in term newborns: a case-control study. Dev Med Child Neurol. (2008) 50:19–24. 10.1111/j.1469-8749.2007.02007.x 18173624

[11] Parker SJ Kuzniewicz M Niki H Wu YW. Antenatal and Intrapartum Risk Factors for Hypoxic-Ischemic Encephalopathy in a US Birth Cohort. J Pediatr. (2018) 203:163–9. 10.1016/j.jpeds.2018.08.028 30270166

[12] Novak CM Eke AC Ozen M Burd I Graham EM. Risk Factors for Neonatal Hypoxic-Ischemic Encephalopathy in the Absence of Sentinel Events. Am J Perinatol. (2019) 36:27–33. 10.1055/s-0038-1639356 29579759

[13] Tann CJ Nkurunziza P Nakakeeto M Oweka J Kurinczuk JJ Were J . Prevalence of bloodstream pathogens is higher in neonatal encephalopathy cases vs. controls using a novel panel of real-time PCR assays. PLoS ONE. (2014) 9:e97259. 10.1371/journal.pone.0097259 24836781 PMC4023955

[14] Tann CJ Martinello KA Sadoo S Lawn JE Seale AC Vega-Poblete M . Neonatal Encephalopathy With Group B Streptococcal Disease Worldwide: Systematic Review, Investigator Group Datasets, and Meta-analysis. Clin Infect Dis. (2017) 65:S173–89. 10.1093/cid/cix662 29117330 PMC5850525

[15] Pacora P Chaiworapongsa T Maymon E Kim YM Gomez R Yoon BH . Funisitis and chorionic vasculitis: the histological counterpart of the fetal inflammatory response syndrome. J Matern Fetal Neonatal Med. (2002) 11:18–25. 10.1080/jmf.11.1.18.25 12380603

[16] Gisslen T Singh G Georgieff MK. Fetal inflammation is associated with persistent systemic and hippocampal inflammation and dysregulation of hippocampal glutamatergic homeostasis. Pediatr Res. (2019) 85:703–10. 10.1038/s41390-019-0330-y 30745569 PMC6435426

[17] Russell NJ Seale AC O'Sullivan C Le Doare K Heath PT Lawn JE . Risk of Early-onset neonatal group B Streptococcal disease with maternal colonization worldwide: systematic review and meta-analyses. Clin Infect Dis. (2017) 65:S152–s159. 10.1093/cid/cix655 29117325 PMC5850448

[18] Hagberg H Mallard C Ferriero DM Vannucci SJ Levison SW Vexler ZS . The role of inflammation in perinatal brain injury. Nat Rev Neurol. (2015) 11:192–208. 10.1038/nrneurol.2015.13 25686754 PMC4664161

[19] Nelson KB Penn AA. Is infection a factor in neonatal encephalopathy? Arch Dis Child Fetal Neonatal Ed. (2015) 100:F8–f10. 10.1136/archdischild-2014-306192 25169244

[20] Fleiss B Tann CJ Degos V Sigaut S Van Steenwinckel J Schang AL . Inflammation-induced sensitization of the brain in term infants. Dev Med Child Neurol. (2015) 57 Suppl 3:17–28. 10.1111/dmcn.12723 25800488

[21] Kendall G Peebles D. Acute fetal hypoxia: the modulating effect of infection. Early Hum Dev. (2005) 81:27–34. 10.1016/j.earlhumdev.2004.10.012 15707712

[22] Hagberg H Gressens P Mallard C. Inflammation during fetal and neonatal life: implications for neurologic and neuropsychiatric disease in children and adults. Ann Neurol. (2012) 71:444–57. 10.1002/ana.22620 22334391

[23] Peebles DM Wyatt JS. Synergy between antenatal exposure to infection and intrapartum events in causation of perinatal brain injury at term. BJOG. (2002) 109:737–9. 10.1111/j.1471-0528.2002.01019.x 12135207

[24] Eklind S Mallard C Arvidsson P Hagberg H. Lipopolysaccharide induces both a primary and a secondary phase of sensitization in the developing rat brain. Pediatr Res. (2005) 58:112–6. 10.1203/01.PDR.0000163513.03619.8D 15879289

[25] Eklind S Mallard C Leverin AL Gilland E Blomgren K Mattsby-Baltzer I . Bacterial endotoxin sensitizes the immature brain to hypoxic–ischaemic injury. Eur J Neurosci. (2001) 13:1101–6. 10.1046/j.0953-816x.2001.01474.x 11285007

[26] Froen JF Amerio G Stray-Pedersen B Saugstad OD. Detrimental effects of nicotine and endotoxin in the newborn piglet brain during severe hypoxemia. Biol Neonate. (2002) 82:188–96. 10.1159/000063610 12373070

[27] Yang L Sameshima H Ikeda T Ikenoue T. Lipopolysaccharide administration enhances hypoxic-ischemic brain damage in newborn rats. J Obstet Gynaecol Res. (2004) 30:142–7. 10.1111/j.1447-0756.2003.00174.x 15009619

[28] Martinello KA Meehan C Avdic-Belltheus A Lingam I Ragab S Hristova M . Acute LPS sensitization and continuous infusion exacerbates hypoxic brain injury in a piglet model of neonatal encephalopathy. Sci Rep. (2019) 9:10184. 10.1038/s41598-019-46488-y 31308390 PMC6629658

[29] Ikeda T Mishima K Aoo N Egashira N Iwasaki K Fujiwara M . Combination treatment of neonatal rats with hypoxia-ischemia and endotoxin induces long-lasting memory and learning impairment that is associated with extended cerebral damage. Am J Obstet Gynecol. (2004) 191:2132–41. 10.1016/j.ajog.2004.04.039 15592303

[30] Mottahedin A Svedin P Nair S Mohn CJ Wang X Hagberg H . Systemic activation of Toll-like receptor 2 suppresses mitochondrial respiration and exacerbates hypoxic-ischemic injury in the developing brain. J Cereb Blood Flow Metab. (2017) 37:1192–8. 10.1177/0271678X17691292 28139935 PMC5453473

[31] Stridh L Mottahedin A Johansson ME Valdez RC Northington F Wang X . Toll-like receptor-3 activation increases the vulnerability of the neonatal brain to hypoxia-ischemia. J Neurosci. (2013) 33:12041–51. 10.1523/JNEUROSCI.0673-13.2013 23864690 PMC3713735

[32] Osredkar D Sabir H Falck M Wood T Maes E Flatebo T . Hypothermia does not reverse cellular responses caused by lipopolysaccharide in neonatal hypoxic-ischaemic brain injury. Dev Neurosci. (2015) 37:390–7. 10.1159/000430860 26087775

[33] Osredkar D Thoresen M Maes E Flatebo T Elstad M Sabir H. Hypothermia is not neuroprotective after infection-sensitized neonatal hypoxic-ischemic brain injury. Resuscitation. (2014) 85:567–72. 10.1016/j.resuscitation.2013.12.006 24361672

[34] Falck M Osredkar D Maes E Flatebo T Wood TR Sabir H . Hypothermic neuronal rescue from infection-sensitised hypoxic-ischaemic brain injury is pathogen dependent. Dev Neurosci. (2017) 39:238–47. 10.1159/000455838 28407632

[35] Chevin M Guiraut C Maurice-Gelinas C Deslauriers J Grignon S Sebire G. Neuroprotective effects of hypothermia in inflammatory-sensitized hypoxic-ischemic encephalopathy. Int J Dev Neurosci. (2016) 55:1–8. 10.1016/j.ijdevneu.2016.09.002 27616300

[36] Chevin M Guiraut C Sebire G. Effect of hypothermia on interleukin-1 receptor antagonist pharmacodynamics in inflammatory-sensitized hypoxic-ischemic encephalopathy of term newborns. J Neuroinflammation. (2018) 15:214. 10.1186/s12974-018-1258-6 30060742 PMC6066954

[37] Martinello KA Meehan C Avdic-Belltheus A Lingam I Mutshiya T Yang Q . Hypothermia is not therapeutic in a neonatal piglet model of inflammation-sensitized hypoxia-ischemia. Pediatr Res. (2021) 1–12. 10.1038/s41390-021-01584-6 34050269 PMC8160560

[38] Pauliah SS Shankaran S Wade A Cady EB Thayyil S. Therapeutic hypothermia for neonatal encephalopathy in low- and middle-income countries: a systematic review and meta-analysis. PLoS ONE. (2013) 8:e58834. 10.1371/journal.pone.0058834 23527034 PMC3602578

[39] Thayyil S Pant S Montaldo P Shukla D Oliveira V Ivain P . Hypothermia for moderate or severe neonatal encephalopathy in low-income and middle-income countries (HELIX): a randomised controlled trial in India, Sri Lanka, and Bangladesh. The Lancet Global Health. (2021) 9:e1273–85. 10.1016/S2214-109X(21)00264-3 34358491 PMC8371331

[40] Mourvillier B Tubach F van de Beek D Garot D Pichon N Georges H . Induced hypothermia in severe bacterial meningitis: a randomized clinical trial. JAMA. (2013) 310:2174–83. 10.1001/jama.2013.280506 24105303

[41] Itenov TS Johansen ME Bestle M Thormar K Hein L Gyldensted L . Induced hypothermia in patients with septic shock and respiratory failure (CASS): a randomised, controlled, open-label trial. Lancet Respir Med. (2018) 6:183–92. 10.1016/S2213-2600(18)30004-3 29325753 PMC10928558

[42] Liberati A Altman DG Tetzlaff J Mulrow C Gotzsche PC Ioannidis JP . The PRISMA statement for reporting systematic reviews and meta-analyses of studies that evaluate health care interventions: explanation and elaboration. J Clin Epidemiol. (2009) 62:e1–34. 10.1016/j.jclinepi.2009.06.006 19631507

[43] Chien PF Khan KS Siassakos D. Registration of systematic reviews: PROSPERO. BJOG. (2012) 119:903–5. 10.1111/j.1471-0528.2011.03242.x 22703418

[44] Chalak L Ferriero DM Gressens P Molloy E Bearer C. A 20 years conundrum of neonatal encephalopathy and hypoxic ischemic encephalopathy: are we closer to a consensus guideline? Pediatr Res. (2019) 86:548–9. 10.1038/s41390-019-0547-9 31450231

[45] Molloy EJ Bearer C. Neonatal encephalopathy versus hypoxic-ischemic encephalopathy. Pediatr Res. (2018) 84:574. 10.1038/s41390-018-0169-7 30214023

[46] Molloy EJ Wynn JL Bliss J Koenig JM Keij FM McGovern M . Neonatal sepsis: need for consensus definition, collaboration and core outcomes. Pediatr Res. (2020) 88:2–4. 10.1038/s41390-020-0850-5 32193517

[47] Fjalstad JW Stensvold HJ Bergseng H Simonsen GS Salvesen B Ronnestad AE . Early-onset sepsis and antibiotic exposure in term infants: a nationwide population-based study in Norway. Pediatr Infect Dis J. (2016) 35:1–6. 10.1097/INF.0000000000000906 26368059

[48] Drageset M Fjalstad JW Mortensen S Klingenberg C. Management of early-onset neonatal sepsis differs in the north and south of Scandinavia. Acta Paediatr. (2017) 106:375–81. 10.1111/apa.13698 27935180

[49] van Laerhoven H de Haan TR Offringa M Post B van der Lee JH. Prognostic tests in term neonates with hypoxic-ischemic encephalopathy: a systematic review. Pediatrics. (2013) 131:88–98. 10.1542/peds.2012-1297 23248219

[50] Lally PJ Montaldo P Oliveira V Soe A Swamy R Bassett P . Magnetic resonance spectroscopy assessment of brain injury after moderate hypothermia in neonatal encephalopathy: a prospective multicentre cohort study. Lancet Neurol. (2019) 18:35–45. 10.1016/S1474-4422(18)30325-9 30447969 PMC6291458

[51] Ouzzani M Hammady H Fedorowicz Z Elmagarmid A. Rayyan-a web and mobile app for systematic reviews. Syst Rev. (2016) 5:210. 10.1186/s13643-016-0384-4 27919275 PMC5139140

[52] Popay J Roberts HM Sowden A Petticrew M Arai L Rodgers M . Guidance on the Conduct of Narrative Synthesis in Systematic Reviews. Lancaster: Institute for Health Research.

[53] Higgins JPT G.S.e. ([updated March 2011]. The Cochrane Collaboration, 2011.). “Cochrane Handbook for Systematic Reviews of Interventions Version 5.1.0”.

[54] Ryan R Hill S. How to GRADE the quality of the evidence. La Trobe University, Melbourne: CCCG (2016). Available online at: http://cccrg.cochrane.org/author-resources.

[55] Rao R Lee KS Zaniletti I Yanowitz TD DiGeronimo R Dizon MLV . Antimicrobial therapy utilization in neonates with hypoxic-ischemic encephalopathy (HIE): a report from the children's hospital neonatal database (CHND). Journal of Perinatology. (2020) 40:70–8. 10.1038/s41372-019-0527-2 31611619

[56] Hakobyan M Dijkman KP Laroche S Naulaers G Rijken M Steiner K . Outcome of infants with therapeutic hypothermia after perinatal asphyxia and early-onset sepsis. Neonatology. (2018) 115:127–33. 10.1159/000493358 30419568

[57] Frank CM Nikkels PG Harteman JC van Haastert IC Benders MJ Koopman-Esseboom C . Placental pathology and outcome after perinatal asphyxia and therapeutic hypothermia. J Perinatol. (2016) 36:977–84. 10.1038/jp.2016.110 27537858

[58] Orrock JE Panchapakesan K Vezina G Chang T Harris K Wang Y . Association of brain injury and neonatal cytokine response during therapeutic hypothermia in newborns with hypoxic-ischemic encephalopathy. Pediatr Res. (2016) 79:742–7. 10.1038/pr.2015.280 26717001 PMC4853239

[59] Mir IN Johnson-Welch SF Nelson DB Brown LS Rosenfeld CR Chalak LF. Placental pathology is associated with severity of neonatal encephalopathy and adverse developmental outcomes following hypothermia. Am J Obstet Gynecol. (2015) 213:849.e841–847. 10.1016/j.ajog.2015.09.072 26408082

[60] Lachapelle J Chen M Oskoui M Ali N Brown R Wintermark P. Placental pathology in asphyxiated newborns treated with therapeutic hypothermia. J Neonatal Perinatal Med. (2015) 8:33–40. 10.3233/NPM-15814068 25766201

[61] Harteman JC Nikkels PG Benders MJ Kwee A Groenendaal F de Vries LS. Placental pathology in full-term infants with hypoxic-ischemic neonatal encephalopathy and association with magnetic resonance imaging pattern of brain injury. J Pediatr. (2013) 163:968–95.e962. 10.1016/j.jpeds.2013.06.010 23891350

[62] Hayes BC Cooley S Donnelly J Doherty E Grehan A Madigan C . The placenta in infants >36 weeks gestation with neonatal encephalopathy: a case control study. Arch Dis Child Fetal Neonatal Ed. (2013) 98:F233–239. 10.1136/archdischild-2012-301992 22791468

[63] Wintermark P Boyd T Gregas MC Labrecque M Hansen A. Placental pathology in asphyxiated newborns meeting the criteria for therapeutic hypothermia. Am J Obstet Gynecol. (2010) 203:579 e571–579. 10.1016/j.ajog.2010.08.024 20851370

[64] Groenendaal F Casaer A Dijkman KP Gavilanes AW de Haan TR ter Horst HJ . Introduction of hypothermia for neonates with perinatal asphyxia in the Netherlands and Flanders. Neonatology. (2013) 104: 15–21. 10.1159/000348823 23615314

[65] Barkovich AJ Hajnal BL Vigneron D Sola A Partridge JC Allen F . Prediction of neuromotor outcome in perinatal asphyxia: evaluation of MR scoring systems. AJNR Am J Neuroradiol. (1998) 19:143–9. 9432172 PMC8337350

[66] Nelson KB Grether JK. Potentially asphyxiating conditions and spastic cerebral palsy in infants of normal birth weight. Am J Obstet Gynecol. (1998) 179:507–13. 10.1016/S0002-9378(98)70387-4 9731861

[67] Jenster M Bonifacio SL Ruel T Rogers EE Tam EW Partridge JC . Maternal or neonatal infection: association with neonatal encephalopathy outcomes. Pediatr Res. (2014) 76:93–9. 10.1038/pr.2014.47 24713817 PMC4062582

[68] Scheidegger S Held U Grass B Latal B Hagmann C Brotschi B . Association of perinatal risk factors with neurological outcome in neonates with hypoxic ischemic encephalopathy. J Matern Fetal Neonatal Med. (2019) 1–8. 10.1080/14767058.2019.1623196 31117854

[69] Sarnat HB Sarnat MS. Neonatal encephalopathy following fetal distress. A clinical and electroencephalographic study Arch Neurol. (1976) 33:696–705. 10.1001/archneur.1976.00500100030012 987769

[70] Wang X Stridh L Li W Dean J Elmgren A Gan L . Lipopolysaccharide sensitizes neonatal hypoxic-ischemic brain injury in a MyD88-dependent manner. J Immunol. (2009) 183:7471–7. 10.4049/jimmunol.0900762 19917690

[71] Yang D Sun YY Bhaumik SK Li Y Baumann JM Lin X . Blocking lymphocyte trafficking with FTY720 prevents inflammation-sensitized hypoxic-ischemic brain injury in newborns. J Neurosci. (2014) 34:16467–81. 10.1523/JNEUROSCI.2582-14.2014 25471584 PMC4252554

[72] Kendall GS Hristova M Horn S Dafou D Acosta-Saltos A Almolda B . TNF gene cluster deletion abolishes lipopolysaccharide-mediated sensitization of the neonatal brain to hypoxic ischemic insult. Lab Invest. (2011) 91:328–41. 10.1038/labinvest.2010.192 21135813

[73] Yang D Sun YY Nemkul N Baumann JM Shereen A Dunn RS . Plasminogen activator inhibitor-1 mitigates brain injury in a rat model of infection-sensitized neonatal hypoxia-ischemia. Cereb Cortex. (2013) 23:1218–29. 10.1093/cercor/bhs115 22556277 PMC3615353

[74] Yang D Sun YY Lin X Baumann JM Dunn RS Lindquist DM . Intranasal delivery of cell-penetrating anti-NF-kappaB peptides (Tat-NBD) alleviates infection-sensitized hypoxic-ischemic brain injury. Exp Neurol. (2013) 247:447–55. 10.1016/j.expneurol.2013.01.015 23353638 PMC4064308

[75] Serdar M Kempe K Rizazad M Herz J Bendix I Felderhoff-Muser U . Early pro-inflammatory microglia activation after inflammation-sensitized hypoxic-ischemic brain injury in neonatal rats. Front Cell Neurosci. (2019) 13:237. 10.3389/fncel.2019.00237 31178702 PMC6543767

[76] Savard A Lavoie K Brochu ME Grbic D Lepage M Gris D . Involvement of neuronal IL-1beta in acquired brain lesions in a rat model of neonatal encephalopathy. J Neuroinflammation. (2013) 10:110. 10.1186/1742-2094-10-110 24007297 PMC3844447

[77] Savard A Brochu ME Chevin M Guiraut C Grbic D Sebire G. Neuronal self-injury mediated by IL-1beta and MMP-9 in a cerebral palsy model of severe neonatal encephalopathy induced by immune activation plus hypoxia-ischemia. J Neuroinflammation. (2015) 12:111. 10.1186/s12974-015-0330-8 26025257 PMC4449972

[78] Ikeda T Yang L Ikenoue T Mallard C Hagberg H. Endotoxin-induced hypoxic-ischemic tolerance is mediated by up-regulation of corticosterone in neonatal rat. Pediatr Res. (2006) 59:56–60. 10.1203/01.pdr.0000191140.87314.ce 16327010

[79] Harding B Conception K Li Y Zhang L. Glucocorticoids protect neonatal rat brain in model of hypoxic-ischemic encephalopathy (HIE). International Journal of Molecular Sciences. (2017) 18. 10.3390/ijms18010017 28025500 PMC5297652

[80] Girard S Sebire H Brochu ME Briota S Sarret P Sebire G. Postnatal administration of IL-1Ra exerts neuroprotective effects following perinatal inflammation and/or hypoxic-ischemic injuries. Brain Behav Immun. (2012) 26:1331–9. 10.1016/j.bbi.2012.09.001 22982341 PMC5023428

[81] Girard S Larouche A Kadhim H Rola-Pleszczynski M Gobeil F Sebire G. Lipopolysaccharide and hypoxia/ischemia induced IL-2 expression by microglia in neonatal brain. Neuroreport. (2008) 19:997–1002. 10.1097/WNR.0b013e3283036e88 18580568

[82] Lehnardt S Massillon L Follett P Jensen FE Ratan R Rosenberg PA . Activation of innate immunity in the CNS triggers neurodegeneration through a Toll-like receptor 4-dependent pathway. Proc Natl Acad Sci USA. (2003) 100:8514–9. 10.1073/pnas.1432609100 12824464 PMC166260

[83] Stigger F Lovatel G Marques M Bertoldi K Moyses F Elsner V . Inflammatory response and oxidative stress in developing rat brain and its consequences on motor behavior following maternal administration of LPS and perinatal anoxia. Int J Dev Neurosci. (2013) 31:820–7. 10.1016/j.ijdevneu.2013.10.003 24140242

[84] Brochu ME Girard S Lavoie K Sebire G. Developmental regulation of the neuroinflammatory responses to LPS and/or hypoxia-ischemia between preterm and term neonates: An experimental study. J Neuroinflammation. (2011) 8:55. 10.1186/1742-2094-8-55 21599903 PMC3121616

[85] Wang X Svedin P Nie C Lapatto R Zhu C Gustavsson M . N-acetylcysteine reduces lipopolysaccharide-sensitized hypoxic-ischemic brain injury. Ann Neurol. (2007) 61:263–71. 10.1002/ana.21066 17253623

[86] Dhillon SK Gunn AJ Jung Y Mathai S Bennet L Fraser M. Lipopolysaccharide-induced preconditioning attenuates apoptosis and differentially regulates TLR4 and TLR7 gene expression after ischemia in the preterm ovine fetal brain. Dev Neurosci. (2015) 37:497–514. 10.1159/000433422 26184807

[87] Lin HY Huang CC Chang KF. Lipopolysaccharide preconditioning reduces neuroinflammation against hypoxic ischemia and provides long-term outcome of neuroprotection in neonatal rat. Pediatr Res. (2009) 66:254–9. 10.1203/PDR.0b013e3181b0d336 19531979

[88] Vexler ZS Mallard C Hagberg H. Positive and negative conditioning in the neonatal brain. Cond Med. (2018) 1:279–93. 31214666 PMC6581457

[89] Hassell KJ Ezzati M Alonso-Alconada D Hausenloy DJ Robertson NJ. New horizons for newborn brain protection: enhancing endogenous neuroprotection. Arch Dis Child Fetal Neonatal Ed. (2015) 100:F541–552. 10.1136/archdischild-2014-306284 26063194 PMC4680177

[90] Thoresen M. Who should we cool after perinatal asphyxia? Semin Fetal Neonatal Med. (2015) 20:66–71. 10.1016/j.siny.2015.01.002 25667126

[91] Jenkins DD Lee T Chiuzan C Perkel JK Rollins LG Wagner CL . Altered circulating leukocytes and their chemokines in a clinical trial of therapeutic hypothermia for neonatal hypoxic ischemic encephalopathy^*^. Pediatr Crit Care Med. (2013) 14:786–95. 10.1097/PCC.0b013e3182975cc9 23897243

[92] Chakkarapani E Davis J Thoresen M. Therapeutic hypothermia delays the C-reactive protein response and suppresses white blood cell and platelet count in infants with neonatal encephalopathy. Arch Dis Child Fetal Neonatal Ed. (2014) 99:F458–463. 10.1136/archdischild-2013-305763 24972990

[93] Martinello KA Meehan C Avdic-Belltheus A Lingam I Mutshiya T Yang Q . Hypothermia is not therapeutic in a piglet model of LPS sensitised neonatal encephalopathy. J Paediatr Child Health. (2019) 55:33–33. 10.1111/jpc.14409_82

[94] Falck M Osredkar D Maes E Flatebo T Wood TR Walloe L . Hypothermia Is Neuroprotective after Severe Hypoxic-Ischaemic Brain Injury in Neonatal Rats Pre-Exposed to PAM3CSK4. Dev Neurosci. (2018) 40:189–97. 10.1159/000487798 29860252

[95] Miller SP Ramaswamy V Michelson D Barkovich AJ Holshouser B Wycliffe N . Patterns of brain injury in term neonatal encephalopathy. J Pediatr. (2005) 146:453–60. 10.1016/j.jpeds.2004.12.026 15812446

[96] Shankaran S McDonald SA Laptook AR Hintz SR Barnes PD Das A . Neonatal magnetic resonance imaging pattern of brain injury as a biomarker of childhood outcomes following a trial of hypothermia for neonatal hypoxic-ischemic encephalopathy. J Pediatr. (2015) 167:987–993.e983. 10.1016/j.jpeds.2015.08.013 26387012 PMC4700815

[97] Kelly LA O'Dea MI Zareen Z Melo AM McKenna E Strickland T . Altered inflammasome activation in neonatal encephalopathy persists in childhood. Clin Exp Immunol. (2021) 205:89–97. 10.1111/cei.13598 33768526 PMC8209598

