# Supplementary material for: Neurological Outcome Following Newborn Encephalopathy With and Without Perinatal Infection: A Systematic Review
Source: Front Pediatr. 2021 Dec 20;9:787804. doi: 10.3389/fped.2021.787804 (PMC8721111; doi:10.3389/fped.2021.787804)
Supplement: Supplementary file 1 [file Data_Sheet_1.doc]

| **Section/topic** | **#** | **Checklist item** | **Section** |
| --- | --- | --- | --- |
| **TITLE** | | |  |
| Title | 1 | Identify the report as a systematic review, meta-analysis, or both. | Title |
| **ABSTRACT** | | |  |
| Structured summary | 2 | Provide a structured summary including, as applicable: background; objectives; data sources; study eligibility criteria, participants, and interventions; study appraisal and synthesis methods; results; limitations; conclusions and implications of key findings; systematic review registration number. | Abstract |
| **INTRODUCTION** | | |  |
| Rationale | 3 | Describe the rationale for the review in the context of what is already known. | Introduction |
| Objectives | 4 | Provide an explicit statement of questions being addressed with reference to participants, interventions, comparisons, outcomes, and study design (PICOS). | Introduction |
| **METHODS** | | |  |
| Protocol and registration | 5 | Indicate if a review protocol exists, if and where it can be accessed (e.g., Web address), and, if available, provide registration information including registration number. | Methods |
| Eligibility criteria | 6 | Specify study characteristics (e.g., PICOS, length of follow-up) and report characteristics (e.g., years considered, language, publication status) used as criteria for eligibility, giving rationale. | Eligibility criteria |
| Information sources | 7 | Describe all information sources (e.g., databases with dates of coverage, contact with study authors to identify additional studies) in the search and date last searched. | Information sources and search strategies |
| Search | 8 | Present full electronic search strategy for at least one database, including any limits used, such that it could be repeated. | Supplementary B |
| Study selection | 9 | State the process for selecting studies (i.e., screening, eligibility, included in systematic review, and, if applicable, included in the meta-analysis). | Study selection |
| Data collection process | 10 | Describe method of data extraction from reports (e.g., piloted forms, independently, in duplicate) and any processes for obtaining and confirming data from investigators. | Data collection and data items |
| Data items | 11 | List and define all variables for which data were sought (e.g., PICOS, funding sources) and any assumptions and simplifications made. | Data collection and data items |
| Risk of bias in individual studies | 12 | Describe methods used for assessing risk of bias of individual studies (including specification of whether this was done at the study or outcome level), and how this information is to be used in any data synthesis. | Risk of bias in individual studies |
| Summary measures | 13 | State the principal summary measures (e.g., risk ratio, difference in means). | Synthesis of results |
| Synthesis of results | 14 | Describe the methods of handling data and combining results of studies, if done, including measures of consistency (e.g., I^2^) for each meta-analysis. | Synthesis of results |

**Supplementary A – PRISMA Checklist**

| **Section/topic** | **#** | **Checklist item** | **Section** |
| --- | --- | --- | --- |
| Risk of bias across studies | 15 | Specify any assessment of risk of bias that may affect the cumulative evidence (e.g., publication bias, selective reporting within studies). | Risk of bias across studies |
| Additional analyses | 16 | Describe methods of additional analyses (e.g., sensitivity or subgroup analyses, meta-regression), if done, indicating which were pre-specified. | Not applicable |
| **RESULTS** | | |  |
| Study selection | 17 | Give numbers of studies screened, assessed for eligibility, and included in the review, with reasons for exclusions at each stage, ideally with a flow diagram. | Study selection |
| Study characteristics | 18 | For each study, present characteristics for which data were extracted (e.g., study size, PICOS, follow-up period) and provide the citations. | Study characteristics |
| Risk of bias within studies | 19 | Present data on risk of bias of each study and, if available, any outcome level assessment (see item 12). | Risk of bias within studies |
| Results of individual studies | 20 | For all outcomes considered (benefits or harms), present, for each study: (a) simple summary data for each intervention group (b) effect estimates and confidence intervals, ideally with a forest plot. | Results of individual studies |
| Synthesis of results | 21 | Present results of each meta-analysis done, including confidence intervals and measures of consistency. | Not applicable |
| Risk of bias across studies | 22 | Present results of any assessment of risk of bias across studies (see Item 15). | Risk of bias across studies |
| Additional analysis | 23 | Give results of additional analyses, if done (e.g., sensitivity or subgroup analyses, meta-regression [see Item 16]). | Not applicable |
| **DISCUSSION** | | |  |
| Summary of evidence | 24 | Summarize the main findings including the strength of evidence for each main outcome; consider their relevance to key groups (e.g., healthcare providers, users, and policy makers). | Summary of evidence |
| Limitations | 25 | Discuss limitations at study and outcome level (e.g., risk of bias), and at review-level (e.g., incomplete retrieval of identified research, reporting bias). | Strength and limitations |
| Conclusions | 26 | Provide a general interpretation of the results in the context of other evidence, and implications for future research. | Conclusion |
| **FUNDING** | | |  |
| Funding | 27 | Describe sources of funding for the systematic review and other support (e.g., supply of data); role of funders for the systematic review. | Cover page |

**Supplementary B - Search strategies for each database**

Search strategy for MEDLINE and CENTRAL

1. “infant, newborn”[Mesh]
2. “infant*”
3. “newborn*”
4. “neonate*”
5. “perinatal”
6. “chorioamnionitis”[Mesh]
7. “chorioamnionitis”
8. “neonatal sepsis”[Mesh]
9. ”sepsis”
10. “infection”
11. “bacteremia”
12. “funisitis”
13. ”fetal vasculitis”
14. "asphyxia neonatorum"[Mesh]
15. "asphyxia*"
16. "encephalopathy"
17. "hypoxia ischemia, brain"[Mesh]
18. "hypoxia ischaemia"
19. "hypoxic ischemia"
20. "hypoxic ischaemia"
21. "hypoxic ischemic"
22. "hypoxic ischaemic"
23. "HIE"
24. #1 OR #2 OR #3 OR #4 OR #5
25. #6 OR #7 OR #8 OR #9 OR #10 OR #11 OR #12 OR #13
26. #14 OR #15 OR #16 OR #17 OR #18 OR #19 OR #20 OR #21 OR #22 OR #23
27. #24 OR #25 OR #26

Search strategy for Embase

1. “newborn”/exp
2. “infant*”
3. “neonate*”
4. “perinatal”
5. “chorioamnionitis”/exp
6. “newborn sepsis”/exp
7. “newborn infection”/exp
8. “bacteremia”
9. “funisitis”
10. ”fetal vasculitis”
11. "newborn hypoxia”/exp
12. “hypoxic ischemic encephalopathy”/exp
13. "hypoxia ischaemia"
14. "hypoxic ischemia"
15. "hypoxic ischaemia"
16. "hypoxic ischemic"
17. "hypoxic ischaemic"
18. "HIE"
19. "asphyxia*"
20. "encephalopathy"
21. #1 OR #2 OR #3 OR #4
22. #5 OR #6 OR #7 OR #8 OR #9 OR #10
23. #11 OR #12 OR #13 #14 OR #15 OR #16 OR #17 OR #18 OR #19 OR #20
24. #21 OR #22 OR #23 AND #24

Search strategy for Web of Science

1. “infant*”
2. “newborn*”
3. “neonate*”
4. “perinatal”
5. “chorioamnionitis”
6. ”sepsis”
7. “infection”
8. “bacteremia”
9. “funisitis”
10. ”fetal vasculitis”
11. "hypoxia ischaemia"
12. "hypoxic ischemia"
13. "hypoxic ischaemia"
14. "hypoxic ischemic"
15. "hypoxic ischaemic"
16. "HIE"
17. "asphyxia*"
18. "encephalopathy"
19. #1 OR #2 OR #3 OR #4
20. #5 OR #6 OR #7 OR #8 OR #9 OR #10
21. #11 OR #12 OR #13 OR #14 OR #15 OR #16 OR #17 OR #18
22. #19 OR #20 OR #21

**Supplementary C - Reasons for exclusion of full-texts**

|  | **Title and authors** | **Reason for exclusion** |
| --- | --- | --- |
| 1 | Potential asphyxiating conditions and spastic cerebral palsy in infants of normal birth weight  Nelson KB, Grether JK | Wrong comparison |
| 2 | Neonatal encephalopathy: association of cytokines with MR spectroscopy and outcome  Bartha AI, Foster-Barber A, Miller SP, Vigneron DB, Glidden DV, Barkovich AJ, Ferriero DM | Wrong comparison |
| 3 | Maternal infection and cerebral palsy in infants of normal birth weight  Grether JK, Nelson KB | Wrong comparison |
| 4 | Antenatal and perinatal conditions correlated to handicap among 4-year-old children  Holst K, Andersen E, Philip J, Henningsen I | Wrong comparison |
| 5 | Management of therapeutic hypothermia for neonatal hypoxic-ischaemic encephalopathy in tertiary centre in South Africa  Kali H, Smit J, Rutherford M | Wrong comparison |
| 6 | Placental pathology in asphyxiated newborns treated with therapeutic hypothermia  Lachapelle J, Chen M, Oskoui M, Ali N, Brown R, Wintermark P | Wrong comparison |
| 7 | The role of fetal inflammatory response syndrome and fetal anemia in nonpreventable term neonatal encephalopathy  Muraskas JK, Kelly AF, Goodman JR, Morrison JC | Wrong comparison |
| 8 | C-reactive protein (CRP) responses in neonates with hypoxic ischaemic encephalopathy  Rath S, Narasimhan R, Lumsden C | Wrong study design |
| 9 | Antenatal risk factors associated with unfavorable neurologic status in newborns and at 2 years of age  Stelmach T, Kallas E, Pisarev H, Talvik T | Wrong comparison |
| 10 | Antecedents of cerebral palsy according to severity of motor impairment  Ahlin K, Himmelmann, Nilsson S, Sengpiel V, Jacobsson B | Wrong study design |
| 11 | Neonatal sepsis: a survey of eight years’ experience at the Louisville General Hospital  Alojipan LC, Andrews BF | Wrong outcome |
| 12 | Neonatal seizures in Nigerian infants  Asindi AA, Antia-Obong OE, Ibia EO, Udo JJ | None-extractable |
| 13 | A snapshot of 1001 children presenting with cerebral palsy to a children’s disability hospital  Banskota B, Shrestha S, Rajbh, Ari T, Banskota AK, Spiegel DA | None-extractable |
| 14 | Cerebral palsy in North Indian children: clinic-etiological profile and co-morbidities  Bhati P, Sharma S, Jain R, Rath B, Beri S, Gupta VK, Aneja S | None-extractable |
| 15 | The burden of hypoxic-ischaemic encephalopathy in Malaysian neonatal intensive care-units  Boo NY, Cheah IG | Wrong outcome |
| 16 | Neurodevelopmental outcome of high risk newborns discharged from a special care baby units in a rural district in India  Chattopadhyah N, Mitra K | Wrong comparison |
| 17 | Chorioamnionitis, maternal fever, and neonatal encephalopathy  Cooke R | Wrong study design |
| 18 | Risk factors for developing epilepsy after neonatal seizures  Da Silva LFG, Nunes ML, Da Costa JC | Wrong comparison |
| 19 | Complications during therapeutic hypothermia after perinatal asphyxia: a comparison with trial data  Diederen CMJ, Van Bel F, Groenendaal F | Wrong outcome |
| 20 | Therapeutic hypothermia for infants with hypoxia ischemic encephalopathy: a five years’ single center experience in Kuwait  Elbahtiti A, Aly NY, Abo-Lila R, Al-Sawan R | Wrong comparison |
| 21 | Inflammatory chemokine expression in the peripheral blood of neonates with perinatal asphyxia and perinatal or nosocomial infections  Fotopoulos S, Mouchtouri A, Xanthou G, Lipsou N, Petrakou E, Xanthou M | Wrong outcome |
| 22 | Chorioamnionitis, cytokines, and brain injury  Freeman JM | Wrong study design |
| 23 | Neonatal brain injuries in England: population-based incidens derived from routinely recorded clinical data held in the National Neonatal Research Database  Gale C, Statnikov Y, Jawad S, Uthaya SN, Modi N | Wrong comparison |
| 24 | Adverse obstetric evens are associated with significant risk of cerebral palsy  Glibert WM, Jacoby BN, Xing G, Danielsen B, Smith LH | Wrong comparison |
| 25 | Neuroimaging of neonatal encephalopathies  Girard N, Confort-Gouny S, Schneider J, Chapon F, Viola A, Pineau | Wrong study design |
| 26 | Sequela of chorioamnionitis  Hagberg H, Wennerholm UB, Savman K | Wrong study design |
| 27 | Prenatal, perinatal, and neonatal risk factors of autism spectrum disorder  Hisle-Gorman E, Susi A, Stokes T, Gorman G, Erdie-Lalena C, Nylund CM | Wrong comparison |
| 28 | The contribution of fetal-newborn complications to motor and cognitive deficits  Low JA, Galbraith RS, Muir DW, Broekhoven LH, Wilkinson JW, Karchmar EJ | Wrong comparison |
| 29 | Neuroprotective body hypothermia among newborns with hypoxic ischemic encephalopathy: three-year experience in a tertiary university hospital. A retrospective observational study  Magalhaes M, Rodrigues FP, Chopard MR, Melo VC, Melhado A, Oliveira, Gallacci CB, Pachi PR, Lima Neto TB | Wrong study design |
| 30 | Elevated total peripheral leukocyte count may identify risk for neurological disability in asphyxiated term neonates  Morkus AA, Hopper AO, Deming DD, Yellon SM, Wycliffe N, Ashwal S, Sowers LC, Peverini RL, Angeles DM | Wrong comparison |
| 31 | Risk factors for neonatal hypoxic-ischemic encephalopathy in the absence of sentinel events  Novak CM, Eke AC, Ozen M, Burd I, Graham REM | Wrong outcome |
| 32 | Neurodevelopmental outcome of ’at risk’ nursery graduates  Paul VK, Radhika S, Deorari AK, Singh M | Wrong comparison |
| 33 | Occult group B streptococcal infection: an important cause of intrauterine asphyxia  Peevy KJ, Chalhub EG | Wrong study design |
| 34 | Outcome of ventilated infants born at term without major congenital abnormalities  Ramadon G, Paul N, Morton M, Peacock JL, Grenough N | Wrong comparison |
| 35 | Elevated circulating fetal nucleated red blood cells and placental pathology in term infants who develop cerebral palsy  Redline RW | Wrong comparison |
| 36 | Placental lesions associated with cerebral palsy and neurologic impairment following term birth  Redline RW, O’Riordan MA | Wrong comparison |
| 37 | Association of perinatal risk factors with neurological outcome in neonates with hypoxic ischemic encephalopathy  Scheidegger S, Held U, Grass B, Latal B, Hagmann C, Brotchi B, Zellinger G, Schulzke SM, Wellman S, Wagner B, Daetwyler K, Bar W, Scharrer B, Tolsa JF, Truttmann A, Schneider J, Pfister RE, Berger TM, Fonatana M, Micallef JP, Hoigne I, Bassler D, Natalucci G, Adams M, Frey B, Bernet V | Wrong outcome |
| 38 | Outcome of newborns with birth asphyxia  Shah GS, Singh R, Das BK | Wrong outcome |
| 39 | Chorioamnionitis and neonatal encephalopathy in term infants with fetal acidemia: histolpathologic correlations  Shalak L, Johnson-Welch S, Perlman JM | Wrong outcome |
| 40 | Ante- and perinatal factors for cerebral palsy: case-control study in Estonia  Stelmach T, Pisarev H, Talvik T | Wrong comparison |
| 41 | Risk factors for cerebral palsy  Suvan S, Kapoor SK, Reddaiah VP, Singh U, Sundaram KR | Wrong comparison |
| 42 | Aetiology of global developmental delay in young children: experience from a tertiary care centre India  Tikaria A, Kabra M, Gupta N, Sapra S, Balakrishnan P, Gulati S, ey RM, Gupta AK | Wrong comparison |
| 43 | New strategies in the diagnosis and treatment of cases of neonatal sepsis and perinatal asphyxia  Torres DBV | Wrong language |
| 44 | Correlation between perinatal risk factors and neurodevelopmental outcomes in children at 24 months of age  Tskimanauri N, Khachapuridze N, Imnadze P, Chanadiri T, Bakhtadze S | None-extractable |
| 45 | Placental pathologu and neonatal brain MRI in a randomized trial of erythropoietin for hypoxic ischemic encephalopathy  Wu YW, Goodman AM, Chang T, Mulkey SB, Gonzalez FF, Mayock DE, Juul SE, Mathur AM, Van Meurs K, Mckinstry RC | Wrong comparison |
| 46 | Maternal or neonatal infection: association with neonatal encephalopathy outcomes  Jenster M, Bonifacio SL, Ruel T, Rogers EE, Tam EW, Partridge JC, Barkovich AJ, Ferriero DM, Glass HC | Wrong comparison |

**Supplementary D - Data extraction template**

| **Review title or ID** |  |
| --- | --- |
| **Study ID** |  |
| **Journal** |  |
| **Country** |  |
| **Date form completed** |  |
| **Person extracting data** |  |
| **Publication type** |  |
| Notes: | |

**General**

|  | **Description as stated in study** | **Location in text** |
| --- | --- | --- |
| Aim of study |  |  |
| Design |  |  |
| Unit of allocation |  |  |
| Duration of participation |  |  |
| Notes: | | |

## Participants

|  | **Description as stated in study** | **Location in text** |
| --- | --- | --- |
| Setting of the study |  |  |
| Inclusion/exclusion criteria for the population |  |  |
| Method of recruiting |  |  |
| Total no. of participants |  |  |
| Characteristics of the population |  |  |
| Notes: | | |

## Exposure and comparators

|  | **Description as stated in study** | **Location in text** |
| --- | --- | --- |
| Group name |  |  |
| No. in group |  |  |
| Inclusion/exclusion criteria for the group |  |  |
| Characteristics of the group |  |  |
| Time of assessment |  |  |
| Notes | | |

## Outcomes

|  | **Description as stated in study** | **Location in text** |
| --- | --- | --- |
| Outcome name |  |  |
| Time points measured |  |  |
| Unit of measurements |  |  |
| Method of measurement |  |  |
| Person measuring/ reporting |  |  |
| Was outcome reported in the protocol or in the methods section? |  |  |
| Notes: | | |

# Data and results

|  | **Description as stated in study** | | | | **Location in text** |
| --- | --- | --- | --- | --- | --- |
| Comparisons |  | | | |  |
| Outcome |  | | | |  |
| No. participant | Exposure | | Control | |  |
|  |  |  |  |  |  |
| Results: | Exposure result | Variance | Control result | Variance |  |
|  |  |  |  |  |  |
|  | Overall results | | Variance | |  |
|  |  |  |  |  |  |
| Results: Table/fig. |  | | | |  |
| Other results |  | | | |  |
| No. missing participants |  | |  | |  |
| Reasons missing |  | |  | |  |
| Unit of analysis |  | | | |  |
| Reanalysis required and possible? |  | | | |  |
| Reanalysis result |  | | | |  |
| Statistical methods used |  | | | |  |
| Notes: | | | | | |

# Other information

|  | **Description as stated in study** | **Location in text** |
| --- | --- | --- |
| Key conclusions |  |  |
| References |  |  |
| Study funding sources |  |  |
| Possible conflicts of interest |  |  |
| Notes: | | |
